# Long-term monitoring reveals an avian species credit in secondary forest patches of Costa Rica

**DOI:** 10.7717/peerj.3539

**Published:** 2017-06-30

**Authors:** Steven C. Latta, Nathan L. Brouwer, Alison Olivieri, Julie Girard-Woolley, Judy F. Richardson

**Affiliations:** 1 National Aviary, Pittsburgh, PA, USA; 2 San Vito Bird Club, San Vito, Coto Brus, Costa Rica; 3 Connecticut Audubon, Fairfield, CT, USA

**Keywords:** Avian abundance, Habitat change, Land use, Neotropical migratory birds, Tropical countryside

## Abstract

Degraded and secondary forests comprise approximately 50% of remaining tropical forest. Bird community characteristics and population trends in secondary forests are infrequently studied, but secondary forest may serve as a “safety net” for tropical biodiversity. Less understood is the occurrence of time-delayed, community-level dynamics such as an extinction debt of specialist species or a species credit resulting from the recolonization of forest patches by extirpated species. We sought to elucidate patterns and magnitudes of temporal change in avian communities in secondary forest patches in Southern Costa Rica biannually over a 10 year period during the late breeding season and mid-winter. We classified birds caught in mist nets or recorded in point counts by residency status, and further grouped them based on preferred habitat, sensitivity to disturbance, conservation priority, foraging guild, and foraging strata. Using hierarchical, mixed-effects models we tested for trends among species that share traits. We found that permanent-resident species increased over time relative to migrants. In both seasons, primary forest species generally increased while species typical of secondary forest, scrub, or edge declined. Species relatively sensitive to habitat disturbance increased significantly over time, whereas birds less sensitive to disturbance decreased. Similarly, generalists with higher habitat breadth scores declined. Because, we found very few changes in vegetation characteristics in secondary forest patches, shifts in the avian community toward primary forest species represent a species credit and are likely related to vegetation changes in the broader landscape. We suggest that natural regeneration and maturation of secondary forests should be recognized as a positive conservation development of potential benefit even to species typical of primary forest.

## Introduction

Most studies of the effects of anthropogenic change on community composition and population trends of tropical birds have focused on the impact of forest fragmentation (Robinson, 1999; Stouffer et al., 2011). Bird communities and population trends in secondary forests are less frequently studied, even though degraded and secondary forests comprise approximately 50% of remaining tropical forest (Chazdon et al., 2009), and are likely to be a dominant feature of tropical landscapes for the foreseeable future (Wright & Muller-Landau, 2006). But because secondary forests display significant differences in structure and composition compared to primary forest (Chazdon, 2003; Lugo & Helmer, 2004), the question of how bird populations respond to the prevalence of secondary forest is of critical importance to conservation biologists.

Secondary forest is generally seen as having reduced vegetation diversity and simplified trophic structure (Chazdon, 2003; Lugo & Helmer, 2004), often resulting in lower avian abundance (Blake & Loiselle, 1991), species richness (Robinson & Terborgh, 1997), and phylogenetic diversity (Frishkoff et al., 2014) relative to primary forest. In the tropics, secondary forest may take many decades or even longer to attain the diversity and structure more typical of primary forest, depending on the nature of the disturbance, soil types, the landscape matrix, and other local factors (Chazdon, 2003; Lugo & Helmer, 2004). But depending on land-use history, secondary successional forest can contribute to a complex mosaic of microhabitats for a variety of species, especially granivorous and frugivorous habitat generalists (Blake & Loiselle, 2001; Şekercioḡlu et al., 2007), and over-wintering Neotropical migrants (Petit et al., 1995). Secondary forest may also provide critical foraging opportunities for some species (Stouffer & Bierregaard, 1995; Levey, 1998; Blake & Loiselle, 2001); frugivores may be especially dependent on spatially and temporally dispersed fruit in secondary forest (Blake & Loiselle, 2001). In contrast, insectivores are more common in mature forests (Blake & Loiselle, 2001) and can be impacted negatively by conversion of primary forests to secondary forests (Stouffer & Bierregaard, 1995; Stratford & Stouffer, 1999; Şekercioḡlu et al., 2002).

While secondary forest may be important in a landscape context, evaluating their potential to serve as a “safety net” for maintaining tropical biodiversity (Wright & Muller-Landau, 2006) is challenged by a paucity of data (Laurance, 2007; Chazdon et al., 2009). Although recent studies in the tropical countryside of Southern Costa Rica have demonstrated the ecological value of forests in agricultural landscapes (Karp et al., 2011; Mendenhall et al., 2011, 2014; Şekercioḡlu et al., 2015), most avian studies have been short-term presence/absence surveys in secondary forest with adjoining primary forest as a baseline. As a result, it is difficult to characterize the dynamics of rare species which make up a substantial part of tropical forest avifaunas (Karr et al., 1990; Terborgh et al., 1990) but are infrequently encountered in short-term studies (Blake & Loiselle, 2000), or to account for inter-annual variation in abundance (Chazdon et al., 2009). Additionally, avifaunal changes may be associated with habitat age, yet existing studies have focused on early successional forests <10 years of age (Chazdon et al., 2009). These shortcomings also limit the possibility of assessing the occurrence of community-level dynamics such as an extinction debt (Tilman et al., 1994; Ford et al., 2009; Jackson & Sax, 2009; Kuussaari et al., 2009), defined as a time-delayed but deterministic extinction of specialists from a focal habitat as the community equilibrates after habitat alteration (Tilman et al., 1994). Even more rarely reported, a time-delayed species credit (Hanski, 2000; Pardini et al., 2010; Lira et al., 2012) may also occur through recolonization of habitat patches by extirpated species.

Evaluations of secondary forest use in the Neotropics also seldom account for seasonality of bird communities associated with altitudinal migration (Stiles, 1988; Loiselle & Blake, 1991; Blake & Loiselle, 2000), or latitudinal migration of long-distance Neotropical migratory birds (Blake & Loiselle, 2001). Loiselle & Blake (1991) found that altitudinal migrants—primarily frugivores and nectarivores—accounted for >30% of local avifauna in Neotropical forests, while Petit et al. (1995) found that insectivorous Neotropical migrants comprise a substantial portion of the over-wintering birds in second-growth habitats. Monitoring of latitudinal migrants on their wintering grounds is also of importance, as the comparison of negative abundance trajectories of over-wintering migrants to stable trends of permanent residents has proven important in developing theories to explain range-wide population declines of a number of Neotropical migratory species (Faaborg et al., 2010).

In this article, we seek to elucidate patterns and magnitudes of temporal change of birds in secondary forests in Southern Costa Rica. We characterize changes in the avian community biannually over a 10 year period during the late breeding season (August) and mid-winter (January). We assess population trends for the most abundant species in forest patches >30 years old, and predict: (1) resident species preferring primary forest will be rare; (2) habitat generalists or those that prefer secondary forests and scrub will be stable or increasing in abundance; (3) insectivores will be declining while frugivores/granivores will increase in abundance; and (4) widely reported declines in Nearctic–Neotropical migratory bird populations will be detectable as declines in our study.

## Methods

### Study sites

This study was conducted on private plots near Las Cruces Biological Station (LCBS), Puntarenas province, Costa Rica (8°47.7N, 82°57.32W). Rainfall at LCBS averages ∼4,000 mm/year. Daytime temperatures range from 13 to 26 °C. The Las Cruces area had relatively intact premontane rainforest until the 1950s when immigration, economic development, and government policies led to deforestation and agricultural production. As a result, annual deforestation rates of 2.1% from 1947 to 1960 and 3.9% from 1960 to 1980 shifted forests to progressively smaller fragments (Zahawi, Duran & Kormann, 2015). Subsequent abandonment of agricultural plots resulted in some regeneration, and these secondary forests are the focus of this study.

**Figure 1:**
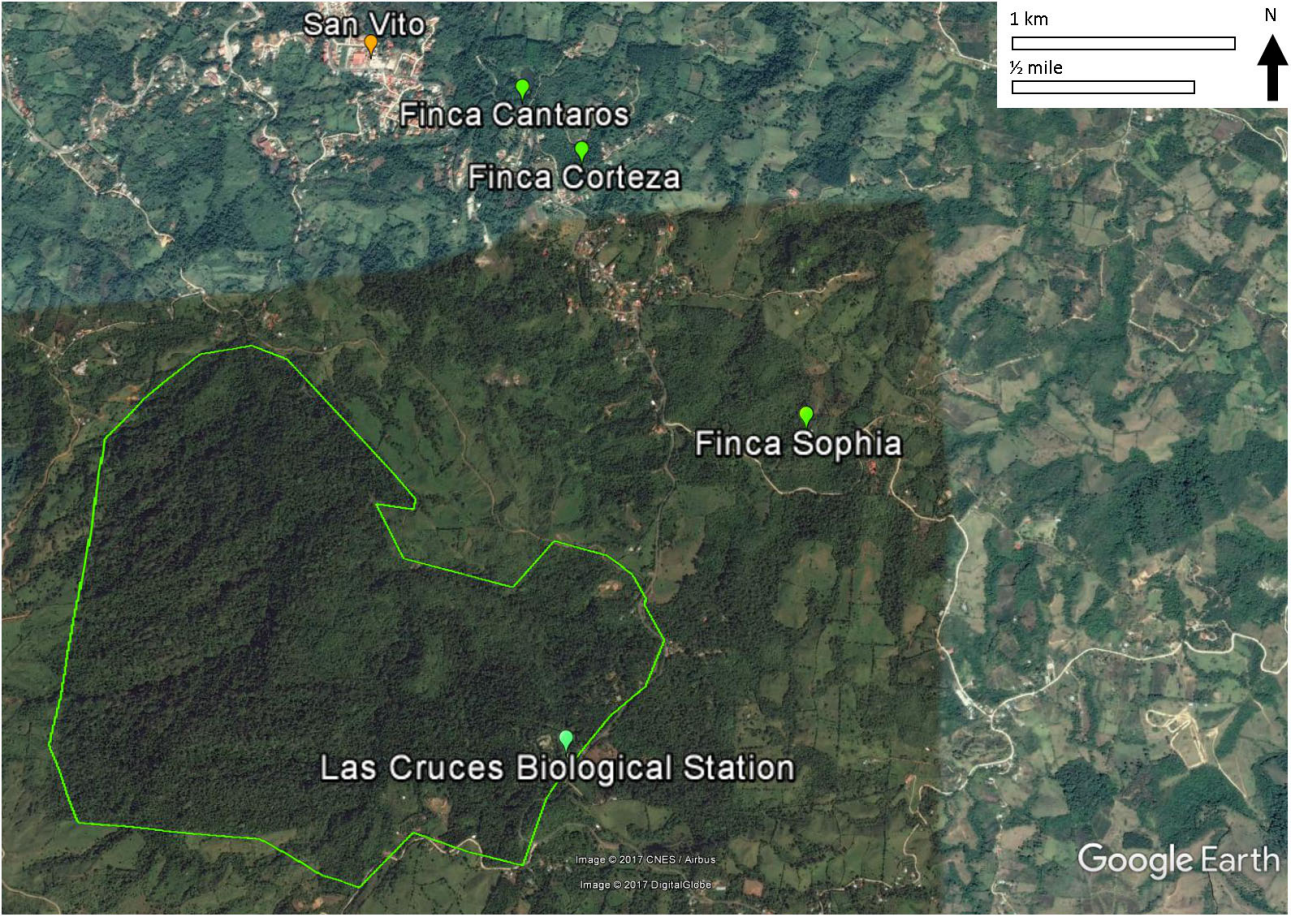
Locations of the three study sites in mature secondary tropical forest, Fincas Sofía, Cántaros, and Corteza, near Las Cruces Biological Station in Puntarenas province, Coto Brus, Costa Rica.

All three monitoring sites were regenerating broadleaf forests >30 years old (Fincas Sofía, Cántaros, and Corteza described in Appendix S1; Fig. 1). Sites were selected based on similarity of vegetation composition and structure. Vegetation was described and quantified by Arce & Brenes (2007) and re-sampled by Pereira (2011) to assess structural and compositional changes. The relevé method, fully described by Ralph et al. (1996) was used, with vegetation data collected in 9–13 variable radius plots (25–50 m) per study site, with each non-overlapping plot centered on a mist net location. Shrub and tree diversity were determined in the plot, and the average height of the lower and upper bounds of the tree stratum (all vegetation ≥5 m) and shrub stratum (all vegetation ≥0.5 and <5 m) were measured. The cover of each stratum was estimated using five cover classes (0–5, 5–25, 25–50, 50–75, and 75–100%). Also for each stratum, the species and their diameter-at-breast-height was measured for the largest and smallest trees in each stratum.

Forest plots did not change dramatically between vegetation sampling periods (Pereira, 2011). In both Fincas Cántaros and Sofía, fewer shrub species were recorded in 2011, with *Miconia* spp. more common in 2011. In addition, canopy closure increased in the tree layer of Finca Cántaros in 2011. Otherwise, there were no significant differences in vegetation diversity, structure, or composition at any of the sites, including the average lower and upper bounds of the vegetation layers, estimated percent cover, and diameter-at-breast-height for the largest and smallest trees in each layer.

### Sampling birds

In tropical forests, point counts tend to be more effective at sampling avifauna in mature forests, while mist nets are more effective in disturbed forests where more species utilize the understory (Blake & Loiselle, 2001). To limit bias, we endeavored to use both methods (Ralph & Scott, 1981; Ralph et al., 1996), sampling biannually 2005–2014 in the late breeding season (July–August, except in 2006 and 2012) and mid-winter (January), although sampling by point counts took place primarily in January (Appendix S2).

We used 15 mist nets (12 × 2.5 m, 30 or 32 mm mesh) in fixed locations at each site, and opened them from 0545 to 1045 on two consecutive days, so net hours were consistent between seasons and among years. All mist-netted birds were identified to species by plumage (Stiles & Skutch, 1989) and to age (juvenile or adult) by plumage or molt limits whenever possible. All birds, except hummingbirds, were banded with a numbered metal band. As capture effort remained constant, we expressed abundance as the number of birds captured/season. Handling of birds was under permissions 720017214 and 221946136 provided by the Ministerio de Recursos Naturales, Energía y Minas of Dirección General de Vida Silvestre (MINAE), and the Institutional Animal Care and Use Committee of the National Aviary and Pittsburgh Zoo/PPG Aquarium permit 2006-SL1.

We conducted 10 min, 50 m fixed-radius audiovisual point counts using the intensive point count protocol of Ralph et al. (1996), with the number of points established (Finca Sofía = 5, Finca Cántaros = 4, Finca Corteza = 2) dependent on the size and shape of each site. Points were placed along narrow foot trails, a minimum of 100 m from other points to help maintain independence. All points were counted once per season and were completed from sunrise—09:30; no counts were conducted in inclement weather. Birds counted from these points were combined into a single mean, so the distance between points is less critical than extensive point counts where each point is intended to be statistically independent (Ralph et al., 1996).

We eliminated fly-overs, and species that are primarily nocturnal, aquatic, or aerial foragers from analyses. We classified birds as permanent residents, latitudinal migrants, or elevational migrants based on AOU (1998), Blake & Loiselle (1991, 2000, 2001), Reid, Harris & Zahawi (2012), and Stiles & Skutch (1989). We assigned species to a single preferred habitat on the basis of Stotz et al. (1996). Habitats were either: (1) primary forest; (2) secondary forest, scrub or edge; or (3) other non-forest habitat. We also classified birds based on habitat breadth (defined as the number of habitats a species occupied across its range), sensitivity to disturbance (designated as high, medium, or low), and conservation priority (scored as 1 or 2 = medium, or 3 or 4 = low), with all data derived from Stotz et al. (1996). Birds were grouped into foraging guild or diet on the basis of principal food items consumed (Stiles & Skutch, 1989; Boyle & Sigel, 2015), and included carnivores, insectivores, frugivores, granivores, nectarivores, and omnivores. We also determined which of four non-mutually exclusive foraging strata were utilized: terrestrial, understory, mid-story, and canopy (Stotz et al., 1996). For some regression analyses, similar groups were pooled to balance factor levels and/or increase sample sizes.

### Characterizing avian communities

We characterized the pool of species using secondary forests by building rarefaction curves from our entire dataset, combining samples across years and sites to increase sample size. We used *iNEXT* (Hsieh, Ma & Chao, 2016) in *R* 3.3.1 (R Core Team, 2016) to compare species richness in different seasons (January, August) for both counts and net captures. For each curve we calculated a Chao 1 non-parametric estimator of richness and Shannon diversity (Chao, 1984; Colwell & Coddington, 1994); we expressed Shannon diversity as the effective number of species (Jost, 2006).

We calculated species richness and Shannon diversity from the raw capture data using *vegan* (Oksanen et al., 2016) in *R*. We modeled changes in ln(richness) and diversity using linear-mixed models (LMMs; Gelman & Hill, 2007). We examined changes in both overall species richness and richness within subgroups (i.e., primary vs secondary forest species).

### Modeling population trends

Using random-slopes Poisson generalized LMMs, we modeled population trends for all species observed during a given season for four or more years. This approach allows for the leveraging of information from groups with larger sample sizes to model rarer species (Gelman & Hill, 2007). An observation-level random effect was also included to correct for over-dispersion (Kéry, 2010). We determined whether trends in net captures depended on species traits by testing for significant year-by-trait and year-trait-season interactions; we modeled each predictor separately to avoid multicollinearity. We calculated the estimated trend for each level of a predictor variable by combining regression parameters and their standard errors (SEs) using the *multcomp* package (Hothorn, Bretz & Westfall, 2008).

To estimate species-specific trends we fit a model without any predictors and extracted species-level slopes (BLUPS; Robins, 1989), and estimated their SEs using the *se.coef* function in the *arm* package (Gelman & Su, 2015). For all models, data from both seasons were used except for those related to latitudinal migration for which only January data was applicable. All models included random-intercepts for year and season nested within year using *lmer* from *lme4* (Bates et al., 2015) in *R*. For both species-richness and population-trend models, we checked whether inclusion of traits and year × trait interactions improved our models by comparing AIC and log-likelihood values to null models with no fixed effects and models with only year as a fixed effect.

## Results

**Figure 2:**
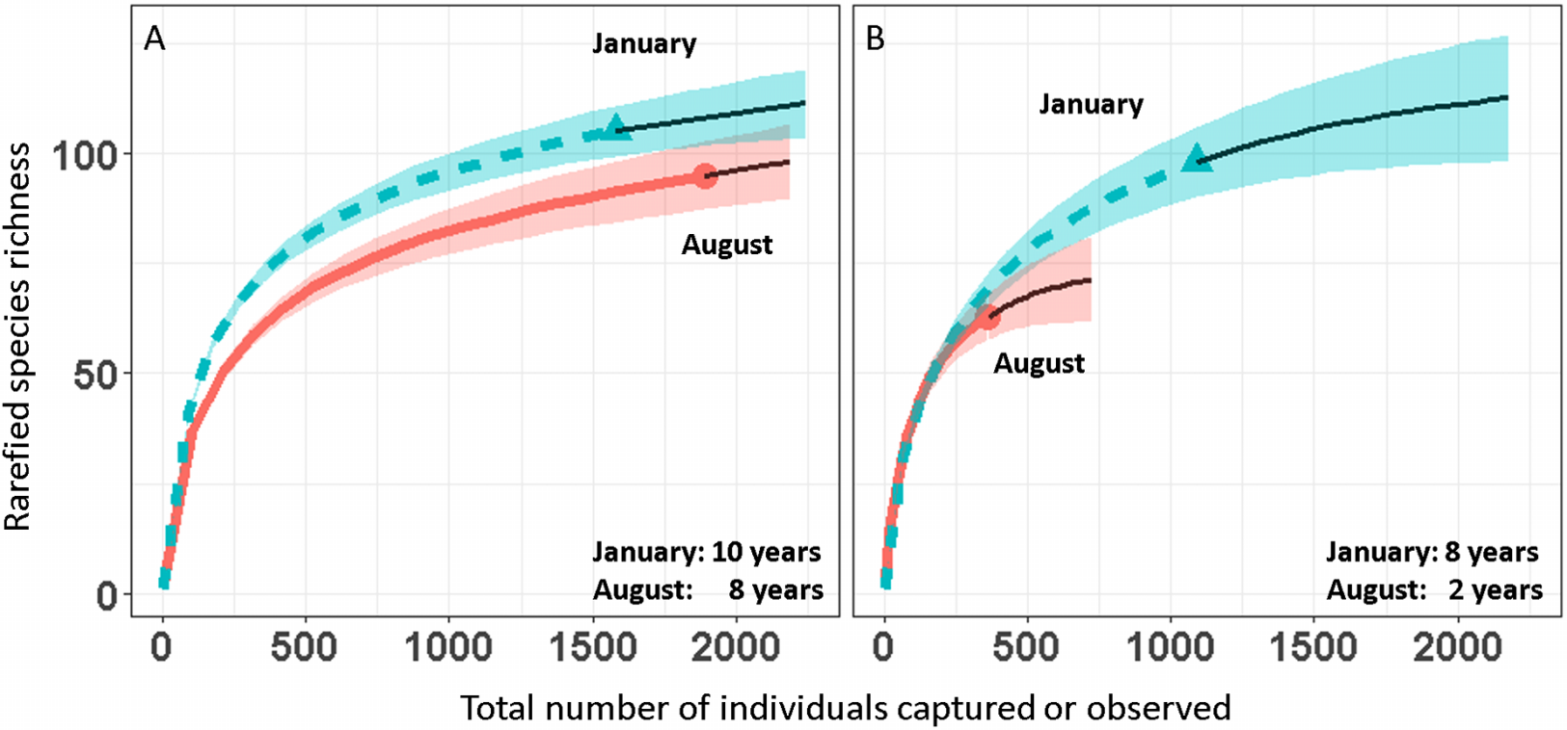
Avian species-accumulation curves for mist-nets (A) and point counts (B). Data are pooled abundances from three secondary forest fragments in Coto Brus, Costa Rica sampled from 2005–2014. Error bands represent 95% CI. Solid black lines represent extrapolated richness.

We report changes in the abundance of birds generated by 1,493 count detections and 3,466 mist-net captures of 152 species of landbirds in secondary forest patches (Table 1). Except for August point counts, species accumulation curves from mist-net (Fig. 2A) and point count (Fig. 2B) data pooled across years approach their asymptotes, indicating that the intensity of sampling was sufficient to characterize the species pool and that few additional species would be added with continued sampling.

**Table 1:** Residency status, ecology, conservation importance, count detections, and mist-net captures of birds in January (mid-winter) and August (late breeding season) in secondary forest fragments of Costa Rica, 2005–2014.

Species	Status^1^	Primary habitat^2^	Forage guild^3^	Sensitivity^4^	Conservation priority^5^	Habitat breadth	Forage strata^6^	Point counts surveys		Mist-net captures	
								Mean ct/pt × 10 January Pt Cts	Mean ct/pt × 10 August Pt Cts	Total January captures	Total August captures
Little Tinamou *Crypturellus soui*	PR	S	O	L	4	3	T	1.1	12.1		
Double-toothed Kite *Harpagus bidentatus*	PR	F	C	M	4	2	C	1.1			
Roadside Hawk *Buteo magnirostris*	PR	S	C	L	4	7	C	13.6	6.1		
Yellow-headed Caracara *Milvago chimachima*	PR	O	O	L	4	4	T–C	4.5	11.4		
Laughing Falcon *Herpetotheres cachinnans*	PR	S	C	L	4	5	C	1.1			
Gray-headed Chachalaca *Ortalis cinereiceps*	PR	S	O	L	4	3	T–C	13.6			
Gray-necked Wood-Rail *Aramides cajanea*	PR	F	O	H	4	4	T	4.5	12.1		
Scaled Pigeon *Patagioenas speciosa*	PR	F	O	M	4	3	C	3.4	26.5		
Short-billed Pigeon *Patagioenas nigrirostris*	PR	F	O	M	4	2	C	2.3			
Ruddy Ground-Dove *Columbina talpacoti*	PR	S	O	L	4	4	T				1
White-tipped Dove *Leptotila verreauxi*	PR	F	O	L	4	5	T–U	29.5	15.2	4	28
Gray-chested Dove *Leptotila cassini*	PR	S	O	M	4	2	T			1	
Ruddy Quail-Dove *Geotrygon montana*	PR	F	O	M	4	3	T				4
Squirrel Cuckoo *Piaya cayana*	PR	F	I	L	4	5	C	6.8	17.4		
White-tipped Sicklebill *Eutoxeres aquila*	PR, EM	F	N	M	4	2	U	4.5		6	7
Bronzy Hermit *Glaucis aeneus*	PR	F	N	H	4	2	U			1	2
Green Hermit *Phaethornis guy*	PR	F	N	M	4	1	U	5.7	9.1	102	150
Stripe-throated Hermit *Phaethornis striigularis*	PR	F	N	M	4	2	U	3.4	3.0	26	43
Purple-crowned Fairy *Heliothryx barroti*	PR	F	N	M	4	2	M–C	2.3		1	
Long-billed Starthroat *Heliomaster longirostris*	PR	S	N	M	4	4	C	1.1		3	1
Garden Emerald *Chlorostilbon assimilis*	PR	S	N	L	4	3	U–C	2.3			5
Scaly-breasted Hummingbird *Phaeochroa cuvierii*	PR, EM	S	N	L	4	3	M–C	11.4		22	33
Violet Sabrewing *Campylopterus hemileucurus*	PR, EM	F	N	M	3	1	U–M		3.0	8	8
White-necked Jacobin *Florisuga mellivora*	PR, EM	F	N	L	4	2	M–C				1
Violet-crowned Woodnymph *Thalurania colombica*	PR, EM	F	N	M	3	4	U–M			3	8
White-tailed Emerald *Elvira chionura*	PR, EM	F	N	M	4	1	U			5	11
Charming Hummingbird *Amazilia decora*	PR, EM	S	N	M	4	2	U–M			2	4
Snowy-bellied Hummingbird *Amazilia edward*	PR	S	N	L	4	3	U–C	6.8		36	13
Rufous-tailed Hummingbird *Amazilia tzacatl*	PR	S	N	L	4	3	U–C	183.0	59.1	231	151
Gartered Trogon *Trogon caligatus*	PR	F	O	M	4	2	M–C	4.5			
Collared Trogon *Trogon collaris*	PR, EM	F	O	M	4	4	M–C	2.3		1	
Blue-crowned Motmot *Momotus coeruliceps*	PR	F	I	M	4	6	U–M	14.8	12.1	8	6
Fiery-billed Aracari *Pteroglossus frantzii*	PR	F	O	M	3	2	C	1.1			1
Black-mandibled Toucan *Ramphastos ambiguus*	PR	F	O	M	3	1	C	5.7	17.4		
Olivaceous Piculet *Picumnus olivaceus*	PR	S	I	L	4	3	M–C			18	5
Red-crowned Woodpecker *Melanerpes rubricapillus*	PR	S	I	L	4	5	C	19.3	12.1	2	
Smoky-brown Woodpecker *Picoides fumigatus*	PR	F	I	L	4	4	M–C			9	3
Golden-olive Woodpecker *Colaptes rubiginosus*	PR	F	I	L	4	5	C				1
Lineated Woodpecker *Dryocopus lineatus*	PR	F	I	L	4	6	C	2.3	8.3		
Slaty Spinetail *Synallaxis brachyura*	PR	S	I	L	4	4	U	5.7		8	7
Buff-throated Foliage-gleaner *Automolus ochrolaemus*	PR	F	I	M	4	2	U	2.3	6.1	14	4
Ruddy Foliage-gleaner *Automolus rubiginosus*	PR	F	I	M	4	2	U			6	7
Plain Xenops *Xenops minutus*	PR	F	I	M	4	2	U–M			14	
Ruddy Woodcreeper *Dendrocincla homochroa*	PR	F	I	H	4	3	U			1	
Olivaceous Woodcreeper *Sittasomus griseicapillus*	PR	F	I	M	4	5	M	1.1		19	17
Wedge-billed Woodcreeper *Glyphorynchus spirurus*	PR	F	I	M	4	2	U–M			8	7
Spotted Woodcreeper *Xiphorhynchus erythropygius*	PR	F	I	M	4	2	M			2	
Streak-headed Woodcreeper *Lepidocolaptes souleyetii*	PR	F	I	L	4	5	U–M	8.0	3.0	4	4
Spot-crowned Woodcreeper *Lepidocolaptes affinis*	PR	F	I	M	4	3	M			1	
Plain Antvireo *Dysithamnus mentalis*	PR, EM	F	I	M	4	2	U–M				2
Slaty Antwren *Myrmotherula schisticolor*	PR	F	I	M	4	2	U			3	5
Black-faced Antthrush *Formicarius analis*	PR	F	I	M	4	2	T	1.1			
Yellow-crowned Tyrannulet *Tyrannulus elatus*	PR	F	I	L	4	4	C			1	3
Greenish Elaenia *Myiopagis viridicata*	PR	F	F	M	4	4	C			3	3
Yellow-bellied Elaenia *Elaenia flavogaster*	PR	S	F	L	4	4	C	15.9	6.1		2
Lesser Elaenia *Elaenia chiriquensis*	LM	S	F	L	4	4	C				1
Olive-striped Flycatcher *Mionectes olivaceus*	PR, EM	F	F	M	4	3	U–C			1	
Ochre-bellied Flycatcher *Mionectes oleagineus*	PR, EM	F	F	M	4	3	U–C	1.1	3.0	30	47
Slaty-capped Flycatcher *Leptopogon superciliaris*	PR	F	I	M	4	1	U–M	1.1			
Paltry Tyrannulet *Zimmerius vilissimus*	PR, EM	F	F	M	4	3	C	92.0	31.8	15	15
Scale-crested Pygmy-Tyrant *Lophotriccus pileatus*	PR	F	I	M	4	2	U–M	11.4	26.5	6	3
Slate-headed Tody-Flycatcher *Poecilotriccus sylvia*	PR	F	I	L	4	5	U			2	7
Common Tody-Flycatcher *Todirostrum cinereum*	PR	S	I	L	4	5	U–C	15.9	3.0	4	
Eye-ringed Flatbill *Rhynchocyclus brevirostris*	PR	F	I	M	4	2	M			10	3
Yellow-olive Flycatcher *Tolmomyias sulphurescens*	PR	F	I	M	4	6	C				1
White-throated Spadebill *Platyrinchus mystaceus*	PR	F	I	M	4	2	U		3.0	6	7
Sulphur-rumped Flycatcher *Myiobius sulphureipygius*	PR	F	I	M	4	3	U–M	1.1		12	11
Bran-colored Flycatcher *Myiophobus fasciatus*	PR	S	I	L	4	3	U				1
Yellow-bellied Flycatcher *Empidonax flaviventris*	LM	F	I	L	4	3	M			11	
Alder Flycatcher *Empidonax alnorum*	LM	S	I	L	4	3	M			1	
Bright-rumped Attila *Attila spadiceus*	PR	F	O	M	4	3	M–C	3.4		4	1
Dusky-capped Flycatcher *Myiarchus tuberculifer*	PR	F	I	L	4	5	M–C	9.1	22.7	1	2
Great Kiskadee *Pitangus sulphuratus*	PR	S	O	L	4	5	T–C	2.3	3.0		
Boat-billed Flycatcher *Megarynchus pitangua*	PR	S	I	L	4	5	C	22.7			1
Social Flycatcher *Myiozetetes similis*	PR	S	O	L	4	4	M–C	1.1			2
Gray-capped Flycatcher *Myiozetetes granadensis*	PR	F	O	L	4	3	C	1.1			4
Piratic Flycatcher *Legatus leucophaius*	LM	S	O	L	4	4	C		3.0		
Tropical Kingbird *Tyrannus melancholicus*	PR	S	I	L	4	5	C	4.5	3.0		
White-ruffed Manakin *Corapipo altera*	PR, EM	F	F	H	4	2	U			42	72
Blue-crowned Manakin *Lepidothrix coronata*	PR	F	F	M	4	2	U–M	1.1		21	16
Orange-collared Manakin *Manacus aurantiacus*	PR	S	F	M	4	2	U	6.8	3.0	17	79
Masked Tityra *Tityra semifasciata*	PR	F	O	M	4	3	C	2.3			
Cinnamon Becard *Pachyramphus cinnamomeus*	PR	S	I	L	4	2	C			2	
White-winged Becard *Pachyramphus polychopterus*	PR	S	I	L	4	4	C				12
Rose-throated Becard *Pachyramphus aglaiae*	PR	F	I	M	4	4	C	1.1		1	
Yellow-throated Vireo *Vireo flavifrons*	LM	S	I	L	4	4	C	10.2		1	
Yellow-green Vireo *Vireo flavoviridis*	LM	S	O	L	4	4	C				1
Lesser Greenlet *Hylophilus decurtatus*	PR	F	I	M	4	4	M–C	4.5			
Rufous-browed Peppershrike *Cyclarhis gujanensis*	PR	S	I	L	4	4	M–C	4.5	6.1		1
Rufous-breasted Wren *Pheugopedius rutilus*	PR	S	I	L	4	2	U–M	75.0	55.3	33	21
Plain Wren *Cantorchilus modestus*	PR	S	I	L	4	3	U	2.3	6.1		
House Wren *Troglodytes aedon*	PR	S	I	L	4	5	U	1.1	11.4	8	7
White-breasted Wood-Wren *Henicorhina leucosticta*	PR	F	I	M	4	2	U	28.4	17.4	24	29
Scaly-breasted Wren *Microcerculus marginatus*	PR	F	I	H	4	1	T–U				1
Orange-billed Nightingale-Thrush *C. aurantiirostris*	PR	S	O	L	4	5	T–U	43.2	12.1	62	59
Swainson’s Thrush *Catharus ustulatus*	LM	F	O	M	4	4	T–U	1.1		4	
Wood Thrush *Hylocichla mustelina*	LM	F	O	M	3	2	T–U			6	
Clay-colored Thrush *Turdus grayi*	PR	S	O	L	4	3	T–M	34.1	135.6	67	195
White-throated Thrush *Turdus assimilis*	PR, EM	F	O	M	4	3	U–M	5.7	12.1	25	51
Ovenbird *Seiurus aurocapilla*	LM	F	I	M	4	2	T–U			33	
Worm-eating Warbler *Helmitheros vermivorum*	LM	F	I	M	3	2	U	2.3		3	
Northern Waterthrush *Parkesia noveboracensis*	LM	F	I	M	4	5	T–U	1.1		35	
Golden-winged Warbler *Vermivora chrysoptera*	LM	S	I	L	3	3	U–M	2.3		1	
Black-and-white Warbler *Mniotilta varia*	LM	F	I	L	4	4	M–C	6.8		16	
Tennessee Warbler *Oreothlypis peregrina*	LM	S	I	L	4	3	C	2.3		10	
Mourning Warbler *Geothlypis philadelphia*	LM	F	I	L	4	3	U	1.1		37	
Kentucky Warbler *Geothlypis formosa*	LM	F	I	M	4	2	U			24	
American Redstart *Setophaga ruticilla*	LM	F	I	L	4	3	M–C	3.4		2	
Tropical Parula *Setophaga pitiayumi*	PR	F	I	M	4	5	C				1
Yellow Warbler *Setophaga petechia*	LM	F	I	L	4	5	U–M			2	
Chestnut-sided Warbler *Setophaga pensylvanica*	LM	F	I	L	4	2	M	76.1		42	
Rufous-capped Warbler *Basileuterus rufifrons*	PR	S	I	L	4	5	U	5.7	6.1	42	21
Buff-rumped Warbler *Myiothlypis fulvicauda*	PR	F	I	M	4	1	T	1.1	25.8	3	13
Wilson’s Warbler *Cardellina pusilla*	LM	S	I	L	4	5	U–M	3.4		25	
Slate-throated Redstart *Myioborus miniatus*	PR	F	I	L	4	3	M–C	9.1	3.0	15	11
Bananaquit *Coereba flaveola*	PR	F	N	L	4	5	M–C	23.9	6.1	28	42
Gray-headed Tanager *Eucometis penicillata*	PR	F	F	M	4	4	U–M			2	7
White-lined Tanager *Tachyphonus rufus*	PR	S	F	L	4	4	U–C			2	1
Cherrie’s Tanager *Ramphocelus costaricensis*	PR	S	F	L	4	3	U–M	44.3	75.8	29	25
Blue-gray Tanager *Thraupis episcopus*	PR	S	F	L	4	4	C	26.1	65.2	3	50
Palm Tanager *Thraupis palmarum*	PR	S	F	L	4	6	C	2.3	8.3		
Silver-throated Tanager *Tangara icterocephala*	PR, EM	F	F	M	4	3	C	42.0	62.9	22	199
Speckled Tanager *Tangara guttata*	PR	F	F	H	3	2	C	5.7	15.2	2	4
Bay-headed Tanager *Tangara gyrola*	PR, EM	F	F	M	4	2	C	6.8		3	4
Golden-hooded Tanager *Tangara larvata*	PR	S	F	L	3	2	C	55.7	39.4	3	8
Scarlet-thighed Dacnis *Dacnis venusta*	PR	F	O	M	4	3	C	1.1	3.0		1
Green Honeycreeper *Chlorophanes spiza*	PR	F	O	M	4	4	C	1.1		1	1
Streaked Saltator *Saltator striatipectus*	PR	S	O	L	4	6	M–C	11.4	19.7	9	19
Buff-throated Saltator *Saltator maximus*	PR	S	O	L	4	3	M–C	47.7	18.2	25	40
Blue-black Grassquit *Volatinia jacarina*	PR	S	O	L	4	7	T–C			8	8
Variable Seedeater *Sporophila corvine*	PR	S	O	L	4	4	U–M	3.4	8.3	46	99
Yellow-bellied Seedeater *Sporophila nigricollis*	PR	S	O	L	4	3	U				1
Ruddy-breasted Seedeater *Sporophila minuta*	PR	O	O	L	4	3	U	2.3			
Thick-billed Seed-Finch *Oryzoborus funereus*	PR	S	O	L	4	3	U–M			1	2
Blue-black Grassquit *Volatinia jacarina*	PR	S	O	L	4	7	T–U		16.7		
Yellow-faced Grassquit *Tiaris olivaceus*	PR	S	O	L	4	2	T–M	27.3	16.7	19	39
Slaty Finch *Haplospiza rustica*	PR, EM	S	F	M	4	2	U–M				1
Chestnut-capped Brush-Finch *Arremon brunneinucha*	PR	F	O	L	4	3	T–U			9	11
Orange-billed Sparrow *Arremon aurantiirostris*	PR	F	O	M	4	1	T	1.1	3.0	19	23
Costa Rican Brush-Finch *Arremon costaricensis*	PR	F	O	H	4	4	T–U			26	21
Black-striped Sparrow *Arremonops conirostris*	PR	S	O	L	4	5	T–U	14.8	9.1	7	8
White-naped Brush-Finch *Atlapetes albinucha*	PR	S	O	M	4	2	T–U			3	1
Common Bush-Tanager *Chlorospingus ophthalmicus*	PR	F	O	M	4	2	U–M			6	2
Summer Tanager *Piranga rubra*	LM	F	F	L	4	1	C	6.8		5	
Red-crowned Ant-Tanager *Habia rubica*	PR	F	O	H	4	1	U–M			15	4
Rose-breasted Grosbeak *Pheucticus ludovicianus*	LM	S	F	L	4	5	C	1.1		2	
Blue-black Grosbeak *Cyanocompsa cyanoides*	PR	F	F	M	4	2	U			4	4
Bronzed Cowbird *Molothrus aeneus*	PR	F	O	L	4	4	T				1
Yellow-billed Cacique *Amblycercus holosericeus*	PR	F	I	M	4	3	C	1.1		1	1
Thick-billed Euphonia *Euphonia laniirostris*	PR	F	F	L	4	4	C	5.7			18
Spot-crowned Euphonia *Euphonia imitans*	PR	F	F	M	3	2	C		9.1	1	4
Lesser Goldfinch *Spinus psaltria*	PR, EM	S	F	L	4	4	C			1	

**Notes:**

1 Status: PR, permanent resident; LM, latitudinal migrant; EM, nomadic or elevational migrant (Blake & Loiselle, 1991, 2000, 2001; Reid, Harris & Zahawi, 2012; Stiles & Skutch, 1989).

2 Primary habitat: F, primary forest; S, secondary forest scrub or edge; O, other non-forest (Stotz et al., 1996).

3 Foraging guild: C, carnivore; I, insectivore; F, frugivore/granivore; N, nectarivore; O, omnivore (Boyle & Sigel, 2015; Stiles & Skutch, 1989).

4 Sensitivity to disturbance: H, high; M, medium; L, low.

5 Conservation priority: 1, urgent; 2, high; 3, medium; 4, low.

6 Foraging strata: T, terrestrial; U, understory; M, mid-story; C, canopy (Stotz et al., 1996).

Over the 10 year study, 95 species were captured in August and 104 in January (Table 1). Point counts detected a similar number of species in January (95) but fewer in August (63). Among all species, 18 were elevational migrants, and of those species recorded in January, 19 were latitudinal migrants (Table 1). The proportions of resident and migratory birds were similar whether calculated by number of species or individuals, and were similar for both mist nests and point counts (Table 2).

**Table 2:** Measures of species richness and diversity from mist-net captures and point counts in January (mid-winter) and August (late breeding season) in secondary forest fragments of Costa Rica, 2005–2014 (NTMB = Neotropical migratory bird).

		January		August	
		Captures	Point counts	Captures	Point counts
Richness (S)	Observed (individuals)	104 (1574)	98 (1089)	95 (1892)	63 (361)
	Chao 1 estimate	121.4	118.8	126.5	74.2
	95% CI	(110.5–153.8)	(105.9–152.7)	(105.5–189.0)	(66.4–100.3)
	Resident species (individuals)	85 (1314)	82 (979)	96 (1892)	63 (361)
	% Resident species (individuals)	81.7 (83.5)	83.7 (89.9)	100 (100)	100 (100)
	NTMB species (individuals)	19 (260)	16 (110)	–	–
	% NTMB species (individuals)	18.3 (16.5)	16.3 (10.1)	–	–
Shannon diversity (H′)	Observed index	3.87	3.65	3.56	3.62
	Effective number of species	47.9	38.6	35.3	37.2
	Chao 1 estimate	49.9	40.9	36.5	41.6
	95% CI	(47.9–53.0)	(38.6–44.4)	(35.3–38.8)	(37.2–46.4)
	Evenness (H′/ln(S))	0.83	0.80	0.78	0.87

Across all years and both seasons, presence/absence data (Table 1) suggest that species preferring primary forest (60.5%) were more common than those preferring secondary forest, scrub or edge (39.5%), but the capture ratio of primary vs secondary forest individuals was closer to 1:1 in January (52.3:47.7%) and August (51.1:48.9%). Among foraging guilds (Table 1), most species were insectivores (41.4%) or omnivores (29.6%), with frugivores (16.4%), nectarivores (10.5%), and carnivores (2.0%) less common. Most species observed during the study were ranked as having low (50.6%) or moderate (44.1%) sensitivity to habitat fragmentation. Nearly all species and individuals recorded in our study sites were also of low conservation priority (Table 1).

Within each year, species richness from mist nets was higher in January (mean = 51.5, SE = 1.4) than in August (46.9, SE = 1.5) due to the influx of migrants (difference between mean = 4.6, SE = 1.6, *χ*^2^_3,4_ = 5.9; *p* = 0.015), though Chao 1 non-parametric estimates of species richness from pooled data from all years showed no difference in the size of the overall August and January species pools (Table 2). Similarly, Shannon diversity from net captures was slightly higher on average in January (mean effective number of species = 33.9, SE = 1.03) than August (mean = 25.6, SE = 1.14; mean difference = 8.3, SE of difference = 1.49; *χ*^2^_3,4_ = 18.7, *p* < 0.0001), while Chao 1 estimates from pooled data were similar (Table 2). Evenness of bird captures was generally high and similar among sites (Table 2) and ranged from 0.78 to 0.87.

### Trends in species richness

**Figure 3:**
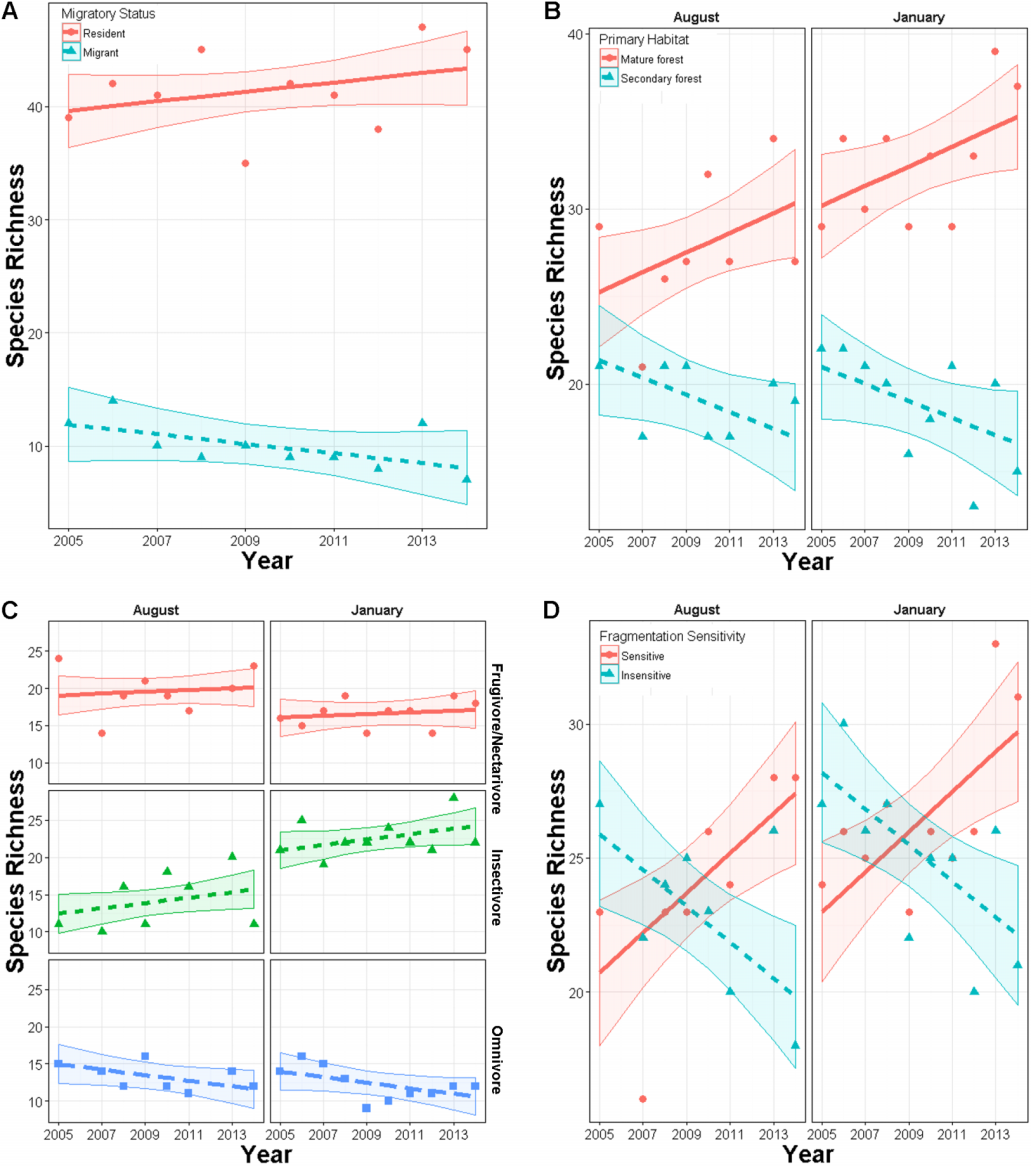
Changes in richness over a 10 year period in secondary forest plots in Costa Rica of: (A) species of permanent residents and Neotropical migratory birds; (B) species which prefer primary forest and those which prefer secondary forest, scrub or edge habitat; (C) species with different diet preferences (frugivores–nectarivores combined with seedeaters, insectivores, and omnivores); (D) species with different sensitivities to habitat degradation. Error bands represent approximately 95% CI.

Total species richness from mist netting did not change over time (year effect *p* = 0.72) in either season (year × season *p* = 0.69). Similarly, Shannon diversity did not change over time overall (*p* = 0.20) or in either season (*p* = 0.50). Within subgroups, species richness did change over time. In January, richness of residents had an upward though non-significant trend (slope = 0.03, SE = 0.04) while richness of latitudinal migrants declined (slope = −0.13, SE = 0.04; year × migrant effect: *χ*^2^_5,6_ = 7.1, *p* = 0.008; Fig. 3A). In both seasons, richness of primary forest species increased marginally over time (slope = 0.052, SE = 0.03), while richness of secondary forest species declined (slope = −0.08, SE = 0.03; year × habitat effect: *χ*^2^_8,9_ = 10.9, *p* < 0.001; Fig. 3B). Species richness within foraging guilds also changed over time (year × foraging guild effect: *p* = 0.015, *χ*^2^_10,12_ = 8.4; Fig. 3C). Richness of omnivores displayed a downward though non-significant trend (slope = −0.10, SE = 0.053) while other guilds were constant (frugivores, nectarivores, and seedeaters: slope = 0.024, SE = 0.04; insectivores: slope = 0.04, SE = 0.052). Richness also changed with respect to disturbance sensitivity (Fig. 3D). When the study began species ranked as moderately to highly sensitive to disturbance were less abundant than those less sensitive to disturbance. However, over time, richness of species highly sensitive to disturbance increased (year × sensitivity effect: *χ*^2^_7,8_ = 26, *p* < 0.00001, slope = 0.087, SE = 0.03), while those with low sensitivity declined (slope = −0.17, SE = 0.024). For these models inclusion of year × trait interaction generally improved model fit by 5–50 AIC units relative to a null model (Appendix S3).

All other potential predictors of trends in species richness were non-significant. Additionally, we did not find any significant season × year interactions, indicating that differences between seasons are due solely to differences in intercept terms of models.

### Abundance trends

Trends in abundance were similar between seasons for all the traits examined; all season × year interactions were non-significant (all *p* > 0.45, Appendix S4), as were all three-way season × year × trait interactions (all *p* > 0.25, Appendix S4). Across seasons, species with different habitat preferences (*p* = 0.001, *χ*^2^_11,12_ = 11.3; Table 3), disturbance sensitivity (*p* = 0.00001, *χ*^2^_11,12_ = 20.14), foraging guilds (*p* = 0.042, *χ*^2^_11,12_ = 4.13), and habitat breadths (*p* = 0.0002, *χ*^2^_8,9_ = 14.25) displayed different trends (Table 3; Appendix S4). Latitudinal migrants in January also differed marginally from residents (*p* = 0.11, *χ*^2^_8,9_ = 2.57). Inclusion of year × trait interactions generally improved the relative fit of the models. For models with *p*-values <0.01, inclusion of the year × trait interaction reduced AIC scores by 8–10 AIC units (Appendix S5). Our foraging guild models have marginal *p*-values and similarly small improvements in AIC (∼1).

**Table 3:** Results of tests for Year × Trait interactions from random-intercepts generalized linear-mixed models.

Trait	Factors levels used	χ^2^	df	p
Migration status	Resident vs migrant	2.57	8,9	0.11
Habitat preference	Secondary vs primary forest	11.30	11,12	0.001**
Sensitivity to disturbance	Medium/high vs low	20.14	11,12	<0.0001**
Conservation priority	Medium vs low	0.41	11,12	0.52
Elevational migrant	Elev. migrant vs non-migrant	1.24	11,12	0.27
Obligate canopy use	Obligate vs facultative canopy use	1.82	11,12	0.18
Canopy use	Obligate/facultative vs no canopy use	0.41	11,12	0.52
Foraging guild-2 levels	Omnivore vs specialist	4.13	11,12	0.042**
Foraging guild-3 levels	Omnivore, frugivore/nectarivore, insectivore	4.94	12,14	0.085*
Habitat breadth		14.25	11,12	0.0002

**Notes:**

When three or more categories existed for a trait we combined similar categories to balance factor levels and increase sample size. Full tables with all model terms are in Appendix S4.

* 0.10 > *p* > 0.05;

** *p* < 0.05.

**Figure 4:**
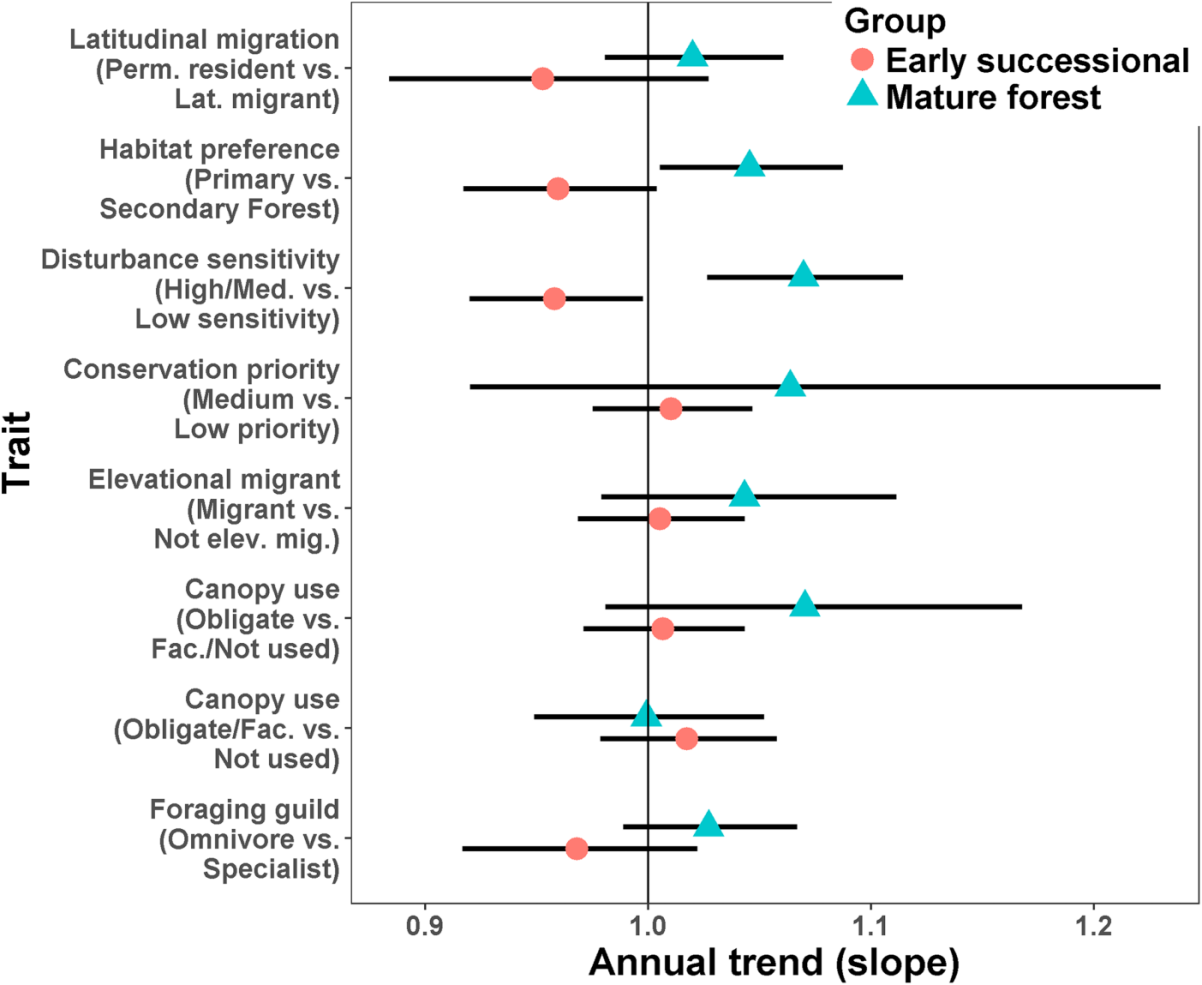
Mean trends in abundance for birds in secondary forest fragments in Costa Rica. Points are transformed slopes from Poisson-normal mixed effects models with species as a random effect, and represent mean changes in abundance for species with different habitat preferences or traits. Error bars are approximately 95% CI.

For a given type of trait, species more characteristic of mature forest generally increased while those more characteristic of secondary forest or early successional habitats tended to decrease (Fig. 4; Appendices S6 and S7). Species preferring primary forest exhibited a positive trend (slope = 0.045, SE = 0.02, *p* = 0.026) while species preferring secondary forest exhibited a marginal decline (slope = −0.041, SE = 0.024, *p* = 0.074). Species ranked as moderately or highly sensitive to disturbance increased over time (slope = 0.067, SE = 0.021, *p* = 0.001) while birds with low sensitivity declined (slope = −0.043, SE = 0.021, *p* = 0.038). Specialist foragers generally increased (slope = 0.027, SE = 0.019) while omnivores decreased (slope = −0.033, SE = 0.024), though neither trend was significantly different from 1 (*p* = 0.17 and 0.23, respectively). Finally, species scored as using more habitats decreased in abundance (slope = −0.035, SE = 0.009, *p* < 0.0001).

### Species-levels trends

Significant species-specific trends were found in either season for five species (Table 4; Appendix S8). Of these, four showed positive population trends and were primary forest species. Only one species (Variable Seedeater) showed a highly significant negative trend, and that species is an omnivore typical of scrub and edge habitat.

**Table 4:** Species in which a significant trend in population size occurred as indicated by mist-net captures in either August (breeding season) or January (mid-winter) in secondary forest fragments of Southern Costa Rica.

Species	August		January	
	Slope	95% CI	Slope	95% CI
Green Hermit	1.12**	1.01–1.24	1.09*	0.99–1.20
Stripe-throated Hermit	1.12**	1.00–1.26	1.03	0.91–1.16
Scaly-breasted Hummingbird	0.97	0.86–1.09	0.92*	0.81–1.05
Violet-crowned Woodnymph	1.10*	0.95–1.27		
Snowy-bellied Hummingbird	0.95	0.83–1.09	1.09*	0.96–1.22
Rufous-tailed Hummingbird	1.08*	0.97–1.19	1.08*	0.98–1.18
Slaty Spinetail	0.92*	0.79–1.07	0.94	0.81–1.09
Buff-throated Foliage-Gleaner	0.99	0.84–1.15	1.10*	0.96–1.27
Ochre-bellied Flycatcher	1.08*	0.97–1.22	0.98	0.87–1.11
Sulphur-rumped Flycatcher	1.11*	0.96–1.28	1.17**	1.01–1.34
White-ruffed Manakin	1.06*	0.95–1.19	0.96	0.86–1.07
White-winged Becard	1.13*	0.98–1.30		
White-breasted Wood-Wren	1.10*	0.97–1.24	1.07	0.94–1.21
Clay-colored Thrush	1.13*	1.02–1.26	0.98	0.88–1.09
White-throated Thrush	1.17**	1.04–1.32	1.04	0.92–1.17
Ovenbird			0.94*	0.84–1.06
Buff-rumped Warbler	1.11*	0.97–1.28		
Wilson’s Warbler			0.90*	0.79–1.02
Bananaquit	0.94	0.84–1.06	0.93*	0.82–1.05
Cherrie’s Tanager	0.94	0.83–1.07	0.90*	0.80–1.02
Silver-throated Tanager	1.00	0.90–1.11	1.07*	0.94–1.21
Blue-black Grassquit	0.87*	0.75–1.02	0.96	0.83–1.12
Variable Seedeater	0.90*	0.81–1.01	0.84**	0.75–0.95
Black-striped Sparrow	0.90*	0.77–1.04		
Red-crowned Ant-Tanager	0.96	0.82–1.12	1.12*	0.98–1.28
Thick-billed Euphonia	1.10*	0.96–1.26		

**Notes:**

** Bold type indicates that the significant trend is >2 SE;

* indicates that the marginally significant trend is >1 SE and <2 SE. Trends with no (*) are not significant; empty cells occur when a species was not observed in a given season. Trend is expressed as average percent change per year, 2005–2014.

Another 19 resident species showed marginally significant population trends (Table 4; Appendix S8). Four species had significant trends in both seasons, and in each case the direction of population change was consistent between seasons. A total of 12 out of 16 species with marginally significant positive population trends were primary forest species, while six of six species with marginally negative trends were associated with secondary forest, scrub or edge. Two additional species with negative trends were over-wintering Neotropical migrants.

## Discussion

We found that over our 10-year study significant changes are occurring in the avian community of secondary forest patches, as we showed that species associated with primary forest are increasing in richness and abundance, while simultaneously, species associated with secondary forest, scrub, or edge habitat, are declining. This pattern may be explained by maturation of the secondary forest at our study sites, suggesting that over the 10-year study, succession progressed sufficiently in these secondary forest plots to allow the development of complex forest structure and microhabitats which are more amenable to species dependent upon primary forest habitats. While intuitively appealing, this explanation does not take into account the fact that our secondary forest plots were all >30 years old, were selected based on their development of a mature forest structure, and repeated vegetation surveys detected few changes in the composition or structure of the sites.

An alternative, non-exclusive hypothesis to explain observed changes in avian species in secondary forest rests more on the vegetation in the surrounding habitat matrix promoting a species credit (Hanski, 2000; Pardini et al., 2010; Lira et al., 2012), in which immigration by species that had been extirpated result in the recolonization of habitat patches. In the case of Coto Brus county where widespread deforestation occurred after 1950, the predicted species credit would be an increase in birds that prefer primary forest habitat, and a decrease in birds that prefer secondary forests, edge or scrub habitat, as we observed. Successful immigration is most likely to occur in landscapes that have undergone intermediate amounts of habitat loss and fragmentation, as habitat connectivity and source populations are required for recolonization of forest patches (Hanski, 2000; Pardini et al., 2010; Lira et al., 2012). In Coto Brus, large blocks of core forest exist at LCBS and the Reserva Indígena Guaymi, and are complemented by a substantial network of linear strips of vegetation (Zahawi, Duran & Kormann, 2015), such as along riparian corridors (Fig. 1).

Lira et al. (2012) showed that the existence of a species credit is related to the amount of forest cover remaining at a landscape scale. Although total forest cover in a 13 km radius around LCBS has been declining, forest loss occurs now at a considerably slower pace than in the 1950–1980s era (Zahawi, Duran & Kormann, 2015), and the rate of forest loss has been largely offset by forest recovery (Zahawi, Duran & Kormann, 2015). New regeneration has contributed to the creation of larger patches of secondary forest, such that 30% of habitat in the LCBS region is classified as secondary forest (Zahawi, Duran & Kormann, 2015). Moreover, across Coto Brus, forest cover has increased over the last 20 years (Zahawi, Duran & Kormann, 2015).

Natural regeneration and maturation of secondary forest can be expected to contribute positively to biodiversity gains since in a landscape setting, a species credit may not just accrue in primary forest fragments, but will also be paid in older secondary forest patches. For example, in studies also from the LCBS region, Şekercioḡlu et al. (2002) concluded that the key to the conservation of understory insectivores inhabiting primary forest in a fragmented landscape lay not in the availability of food in small forest fragments, but in the condition of the country-side habitat surrounding the fragments. This suggests that maturation of secondary forest is contributing to a species credit and the increase of primary forest species, including understory insectivores such as Buff-rumped Warbler, Buff-throated Foliage-Gleaner, and White-breasted Wood-Wren, as found in this study.

While permanent-resident species generally increased over this 10 year period, over-wintering migrants decreased in richness (Fig. 3A) and abundance (Appendix S4). In previous studies of population trends of migrants on their wintering grounds, negative abundance trajectories have been contrasted with stable populations of permanent residents to raise conservation concerns for Neotropical migrants (Faaborg et al., 2010). In this study, only the Ovenbird and Wilson’s Warbler declined significantly (Table 4; Appendix S8). Because the primary habitat occupied by these species’ includes both primary forest (Ovenbird) and secondary forest or scrub (Wilson’s warbler), and because habitat change has occurred in the landscape matrix, it is difficult to generalize as to potential causes of declines in over-wintering migrants. Further analyses of over-winter site persistence and annual return rates for these species would be informative (Faaborg et al., 2010), and would help to distinguish between breeding ground and wintering ground effects.

### Study limitations

While we have demonstrated the value of older secondary forest patches to birds, a better understanding of population health requires investigation of avian vital rates. Even with long-term studies such as this taking into account rare species, inter-annual variation, and seasonality, abundance data alone can be a misleading indicator of population size and habitat quality (Van Horne, 1983). Furthermore, abundance cannot generally be equated with survival or productivity, so data on these demographic parameters are required to assess the quality of secondary forest habitat to these birds.

This level of analysis has seldom been accomplished for species in secondary tropical forest (Barlow et al., 2007). Only recently, Şekercioḡlu et al. (2007) determined nesting success of three avian species in Costa Rica in a landscape including secondary forest fragments. They showed the conservation value of the agricultural countryside and suggested that this can be enhanced with even a modest increase in tree cover in the landscape matrix. Ruiz-Gutierrez, Gavin & Dhondt (2008) used mark-recapture analyses to show that apparent survival of the White-ruffed Manakin (*Corapipo altera*) was lower in primary forest fragments than in the large forest at LCBS, but emphasized the need for population-level studies of other species to test for sources of mortality in forest fragments and surrounding matrix habitats. Assessing survival and population trends is particularly challenging though, because of the need for sampling populations on an annual or more frequent basis using standardized protocols, and this is seldom done (Latta, Ralph & Geupel, 2005; Blake & Loiselle, 2015).

Finally, it should be remembered that other factors extrinsic to forest patches or the landscape matrix may also be affecting local birds—although in general these impacts are expected to be negative. In particular, declines and even extirpations of bird populations in tropical areas have been attributed to changes associated with global warming (Latta et al., 2011; Blake & Loiselle, 2015). While a changing climate may be affecting bird populations at our study sites, it is not likely responsible for the gains in forest-associated species recorded in this article.

### Conservation implications

These results support the importance of secondary forest patches for bird conservation, and emphasize the value of the vegetation in the surrounding habitat matrix. Because we found very few changes in vegetation characteristics of our older secondary forest plots, we suggest that observed changes in the avian community, resulting in a species credit of birds associated with primary forest habitat, are related to changes in vegetation in the broader landscape. As such, we do not suggest that secondary forest patches serve as a safety net per se for tropical biodiversity; in this landscape, the safety net is likely found in the large blocks of core forest where species associated with primary forest persist as source populations. Rather, we suggest that bird diversity increases in maturing secondary forest through a species credit reflecting immigration of primary forest species from these source populations.

Although Zahawi, Duran & Kormann (2015) warned of the continuing threat of an extinction debt in the Las Cruces landscape resulting in the extirpation of additional species, our study suggests that the secondary forests in the tropical countryside are contributing to increasing trends in richness and abundance of bird species associated with primary forest. These results support understandings gained from regional studies that have shown that in landscapes such as Coto Brus, where low-intensity agriculture is a significant part of the land-use matrix, forested riparian corridors (Şekercioḡlu et al., 2015), clusters of trees as small as 20 m wide (Mendenhall et al., 2011), as well as secondary forest patches, can all contribute to biodiversity (Mendenhall et al., 2014), and affect resilience, stability, and ecosystem services (Karp et al., 2011).

Recognition of the value of secondary forests to birds, and perhaps other wildlife (Mendenhall et al., 2014), may impact decision-making on the value of acquiring and protecting secondary forests for conservation planning in these landscapes. This is not to suggest that conservation measures should not be taken to reverse the continuing loss of primary forest in the tropical countryside. While this study offers hope that in some landscapes, maturing secondary forest can provide habitat for a number of primary forest bird species, it should be remembered that secondary forests may differ systematically in vegetation composition and forest structure from the original primary forests (Chazdon, 2003; Lugo & Helmer, 2004), and successional trajectories are affected strongly by initial conditions and the surrounding landscape (Chazdon, 2003; Chazdon et al., 2009). As a result, not all birds associated with primary forests will benefit equally in these landscapes.

## Supplemental Information

10.7717/peerj.3539/supp-1 Supplemental Information 1 Site descriptions.

10.7717/peerj.3539/supp-2 Supplemental Information 2 Dates of sampling by mist nets and point counts.

10.7717/peerj.3539/supp-3 Supplemental Information 3 Log-likelihoods and AIC values for multi-level models on species richness.

10.7717/peerj.3539/supp-4 Supplemental Information 4 Significance tests for nested hierarchical models of species traits and abundance.

10.7717/peerj.3539/supp-5 Supplemental Information 5 Log-likelihoods and AIC values for multilevel models of trends in net captures.

10.7717/peerj.3539/supp-6 Supplemental Information 6 Slopes of trends for species with different traits.

10.7717/peerj.3539/supp-7 Supplemental Information 7 Regression coefficients for models plotted in Fig. 4.

10.7717/peerj.3539/supp-8 Supplemental Information 8 Species-level trends in net captures.

## Additional Information and Declarations

### Field Study Permissions

The following information was supplied relating to field study approvals (i.e., approving body and any reference numbers): Permissions were provided by the Ministerio de Recursos Naturales, Energía y Minas of Dirección General de Vida Silvestre (MINAE).

### Data Availability

The following information was supplied regarding data availability: Raw data and code are available from the Harvard Dataverse: Latta, Steven C.; Brouwer, Nathan L., 2017, “Replication Data for: Long-term monitoring reveals an avian species credit in secondary forest patches of Costa Rica”, DOI 10.7910/DVN/CEVZPL, Harvard Dataverse, V1, UNF:6:L4/PXRZ/xhPr63KA0Ny0aw== https://dataverse.harvard.edu/dataset.xhtml?persistentId=doi:10.7910/DVN/CEVZPL.
