# Supplementary material for: Long-term monitoring reveals an avian species credit in secondary forest patches of Costa Rica
Source: PeerJ. 2017 Jun 30;5:e3539. doi: 10.7717/peerj.3539 (PMC5494173; doi:10.7717/peerj.3539)
Supplement: Supplemental Information 1 [file peerj-05-3539-s001.docx]

Supporting Information, Appendix S1: Site descriptions

*Finca Sofia* is a 13.5-ha reserve (08º 47.7’N, 82º 56.9’W; elevation 1,260 m). Formerly pasture and sugar cane, the regenerating forest was ~30-yr old at initiation of our study. The site also includes two streams bordered by older forest in a narrow riparian zone. The shrub layer, consisting of 9 species, ranged from 0.7-5.8 m, with *Piper sp*., *Miconia sp*., and *Palicourea padifolia* the most prevalent. The arboreal layer was composed of 15 species, with heights ranging from 4.4-30.0 m and DBH of 2.9-35.3 cm. This layer was dominated by *Cecropia* *obtusifolia* and *Palicourea padifolia*, but rainbow eucalyptus (*Eucalyptus deglupta*), amarillon (*Terminalia amazonia*), walnut (*Juglans neotropica*), ceibo (*Erythrina cristagalli*), citrus (*Citrus spp*.), banana (*Musa spp.*), and bamboo (Bambuseae) were also present.

*Finca Cántaros* is a 10.1-ha nature reserve located at 08º 48.6’N, 82º 57.6’W at 1,190 m elevation, and is characterized by a well-developed over story and thick undergrowth surrounding a small pond. The shrub layer, ranging from 0.4-6.0 m, was represented by 13 species with *Miconia trinervia*, *Cecropia* *obtusifolia*, and *Miconia sp*. most abundant. The arboreal layer included 13 species with *Virola koschnyi* and *Psidium guajaba* dominant. Mean height ranged from 4.9-27.0 m, and DBH was 3.9-81.8 cm; canopy cover was 50-75%.

*Finca Corteza* is a 4.9-ha reserve at 1,280 m elevation (08º 48.5’N, 82º 57.5’W). Finca Corteza was formerly a coffee plantation abandoned ~45 yr ago and now has established second-growth forest surrounding a seasonal stream. The shrub layer was represented by 12 species with *Piper sp*. and *Miconia trinervia*, and *Dendropanax costaricense* most common. Mean shrub heights ranged from 0.4-5.1 m. The arboreal layer was dominated by *Cecropia* *obtusifolia* and *Miconia* *trinervia*, but 11 other species were recorded, including *Terminalia amazonia*, corteza (*Tabebuia aurea*), and *Citrus spp*. Mean heights of trees ranged from 5.0-32.0 m, and mean DBH values ranged 3.1-72.1 cm. Canopy cover was 25-50%.
