# Supplementary material for: Long-term monitoring reveals an avian species credit in secondary forest patches of Costa Rica
Source: PeerJ. 2017 Jun 30;5:e3539. doi: 10.7717/peerj.3539 (PMC5494173; doi:10.7717/peerj.3539)
Supplement: Supplemental Information 2 [file peerj-05-3539-s002.docx]

Supporting Information, Appendix S2.

Dates of sampling by mist nets and point counts in secondary forest. Dates in italicized font indicate that only mist netting was done.

Year Late-breeding season dates Mid-winter dates

2005 *29 June – 4 July* *8-14 January*

2006 *3-9 January*

2007 *16-19 July* 4-9 January

2008 *8-13 August* 12-17 January

2009 *4-9 August* 12-17 January

2010 *1-6 August* 21-26 January

2011 *7-12 August* 16-21 January

2012 22-27 January

2013 14-19 August 13-18 January

2014 19-24 August 12-17 January
