# Supplementary material for: Long-term monitoring reveals an avian species credit in secondary forest patches of Costa Rica
Source: PeerJ. 2017 Jun 30;5:e3539. doi: 10.7717/peerj.3539 (PMC5494173; doi:10.7717/peerj.3539)
Supplement: Supplemental Information 3 [file peerj-05-3539-s003.docx]

Supporting Information, Appendix S3

Log-likelihoods and AIC values for multilevel models on log-transformed species richness values for subgroups of species with different traits. There are two subsets of models: those that used data from both August and January, and one that only used data from January. Models that included both August and January data are of the form:

Null: R = **β_Int_.**

Year: R = **β_Int_.+ β_Year_***Year

Year+Trait: R = **β_Int._+ β_Trait_***Trait_SF_ **+ β_Jan_***Jan._Yes/No_ **+ β_Year_***Year

Year*Trait: R = **β_Int_.+ β_Trait_***Trait_SF_ **+ β_Jan_***Jan._Yes/No_ **+ β_Year_***Year **+ β_Year_*trait *Year***Trait_SF_

Where R is the number of species captured in mist nest, Trait_SF_ is a 0/1 indicator for factor levels typical of primary (0) or secondary (1) forest (SF). Jan._Yes/No_ is an indicator variable for net captures in August (0) or January (1). Since latitudinal migrants occur only in January, these model lacked the **β_Jan_ *** Jan._Yes/No_ term. For both subsets of models with report AIC and log likelihoods for null models with no fixed effects and models with just year as a predictor to represent an overall trend for all species. For each trait we then report AIC and log likelihoods for the focal model with the Year*Trait interaction and a nested model that lacks this interaction (Year+Trait). We then report the AIC of a given model minus AIC of the appropriate null model (AIC-AIC_null_) to gauge whether inclusion of a trait in a model improves the model; negative values indicate improvements in the fit of the model relative to the null. P-values from Table 3 for the focal Year*trait interaction are also shown for reference.

| **Months** | **Trait** | **Model type** | **df** | **log Likelihood** | **AIC** | **AIC-AIC_null_** | **p-values** |
| --- | --- | --- | --- | --- | --- | --- | --- |
| **January** | Null model (no predictors) | null | 3 | -23.1 | 52.2 |  |  |
|  | Year model | year | 4 | -23.9 | 55.8 | -4.6 |  |
|  |  |  |  |  |  |  |  |
|  | Migration status | year+trait | 8 | 3.8 | 2.4 | 48.8 |  |
|  |  | year*trait | 9 | 4.9 | 2.1 | 49.1 | 0.008 |
|  |  |  |  |  |  |  |  |
| **August and January** | Null model (no predictors) | null | 4 | -7.2 | 22.4 |  |  |
|  | Year model | year | 5 | -9.3 | 28.6 | 6.2 |  |
|  |  |  |  |  |  |  |  |
|  | Habitat preference | year+trait | 6 | 12.9 | -13.9 | -36.3 |  |
|  |  | year*trait | 7 | 13.6 | -13.1 | -35.5 | <0.001 |
|  | Sensitivity to disturbance | year+trait | 6 | 21.8 | -27.6 | -50.0 |  |
|  |  | year*trait | 7 | 12.4 | -10.9 | -33.3 | <0.0001 |
|  | Foraging guild-3 levels | year+trait | 10 | 8.3 | 3.3 | -19.1 |  |
|  |  | year*trait | 12 | 7.8 | 8.3 | -14.1 | 0.015 |
