# Supplementary material for: Long-term monitoring reveals an avian species credit in secondary forest patches of Costa Rica
Source: PeerJ. 2017 Jun 30;5:e3539. doi: 10.7717/peerj.3539 (PMC5494173; doi:10.7717/peerj.3539)
Supplement: Supplemental Information 4 [file peerj-05-3539-s004.docx]

Supporting Information, Appendix S4

Significant tests for nested hierarchical models to determine links between species traits and changes in abundance. The row of the Year*Trait interaction of each model is in grey. (**) indicates p<0.05; (*) indicates 0.1> p>0.05.

| **Trait** | **Test** | **χ^2^** | **df** | **p** |  |
| --- | --- | --- | --- | --- | --- |
| **Migration status** (resident vs. migrant) | Migration status | 0.19 | 7,8 | 0.66 |  |
|  | Year | 0.17 | 7,8 | 0.73 |  |
|  | Year*Migrant or resident | 2.57 | 8,9 | 0.11 |  |
|  |  |  |  |  |  |
| **Habitat preference** (secondary vs. primary forest) | Habitat preference | 5.66 | 10,11 | 0.017 | ****** |
|  | Month | 1.67 | 10,11 | 0.20 |  |
|  | Year | 0.32 | 10,11 | 0.57 |  |
|  | Year*Habitat reference | 11.30 | 11,12 | 0.001 | ****** |
|  | Month*Habitat preference | 0.22 | 12,13 | 0.64 |  |
|  | Month*Year | 0.26 | 13,14 | 0.61 |  |
|  | Month*Year*Habitat pref. | 0.08 | 14,15 | 0.78 |  |
|  |  |  |  |  |  |
| **Sensitivity to disturbance**  (low vs. med/high) | Dist. sensitivity | 3.03 | 10,11 | 0.082 | ***** |
|  | Month | 1.92 | 10,11 | 0.17 |  |
|  | Year | 0.32 | 10,11 | 0.571 |  |
|  | Year*Dist. sensitivity | 20.14 | 11,12 | 0.00001 | ****** |
|  | Month*Dist. sensitivity | 0.45 | 12,13 | 0.50 |  |
|  | Month*Year | 0.15 | 13,14 | 0.70 |  |
|  | Month*Year*Dist. sensitivity | 0.01 | 14,15 | 0.94 |  |
|  |  |  |  |  |  |
| **Conservation priority** | Cons. priority | 1.98 | 10,11 | 0.16 |  |
|  | Month | 1.99 | 10,11 | 0.16 |  |
|  | Year | 0.41 | 10,11 | 0.52 |  |
|  | Year*Cons. priority | 0.41 | 11,12 | 0.52 |  |
|  | Month*Cons. priority | 0.01 | 12,13 | 0.92 |  |
|  | Month*Year | 0.13 | 13,14 | 0.71 |  |
|  | Month*Year*Cons. priority | 1.29 | 14,15 | 0.26 |  |
|  |  |  |  |  |  |
| **Elevational  migrant** | Elev. mig | 0.04 | 10,11 | 0.85 |  |
|  | Month | 2.01 | 10,11 | 0.16 |  |
|  | Year | 0.41 | 10,11 | 0.52 |  |
|  | Year*Elev. mig | 1.24 | 11,12 | 0.27 |  |
|  | Month*Elev. mig | 5.09 | 12,13 | 0.024 | ****** |
|  | Month*Year | 0.13 | 13,14 | 0.71 |  |
|  | Month*Year*Elev. mig | 0.71 | 14,15 | 0.40 |  |

Supporting Information, Appendix S4 continued:

| **Canopy Use** (Facultative vs. Obligate) | Canopy obligate | 0.56 | 10,11 | 0.46 |
| --- | --- | --- | --- | --- |
|  | Month | 2.14 | 10,11 | 0.14 |
|  | Year | 0.40 | 10,11 | 0.53 |
|  | Year*Canopy obligate | 1.82 | 11,12 | 0.18 |
|  | Month*Canopy obligate | 5.22 | 12,13 | 0.022 |
|  | Month*Year | 0.03 | 13,14 | 0.86 |
|  | Month*Year*Canopy ob. | 0.72 | 14,15 | 0.40 |
|  |  |  |  |  |
| **Canopy Use**  (Use vs. Non-use) | Canopy use | 0.05 | 10,11 | 0.82 |
|  | Month | 1.97 | 10,11 | 0.16 |
|  | Year | 0.42 | 10,11 | 0.52 |
|  | Year*Canopy use | 0.41 | 11,12 | 0.52 |
|  | Month*Canopy use | 0.08 | 12,13 | 0.78 |
|  | Month*Year | 0.17 | 13,14 | 0.68 |
|  | Month*Year*Canopy use | 0.80 | 14,15 | 0.37 |
|  |  |  |  |  |
| **Foraging guild-2** (2 levels: Omnivore vs. Specialist) | Forage guild | 1.80 | 10,11 | 0.18 |
|  | Month | 1.88 | 10,11 | 0.17 |
|  | Year | 0.38 | 10,11 | 0.54 |
|  | Year*Forage guild | 4.13 | 11,12 | 0.042 |
|  | Month*Forage guild | 2.65 | 12,13 | 0.104 |
|  | Month*Year | 0.24 | 13,14 | 0.62 |
|  | Month*Year*Forage guild | 0.00 | 14,15 | 1.000 |
|  |  |  |  |  |
| **Foraging guild-3** (3 levels: Omnivore, Frug./Nectarivore, Insectivore) | Forage guild | 6.01 | 10,12 | 0.050 |
|  | Month | 1.13 | 11,12 | 0.29 |
|  | Year | 0.36 | 11,12 | 0.55 |
|  | Year*Forage guild | 4.94 | 12,14 | 0.085 |
|  | Month*Forage guild | 12.28 | 14,16 | 0.002 |
|  | Month*Year | 0.49 | 16,17 | 0.48 |
|  | Month*Year*Forage guild | 0.23 | 17,19 | 0.89 |
|  |  |  |  |  |
| **Habitat breadth**  (Continuous variable) | Habitat breadth | 0.03 | 10,11 | 0.85 |
|  | Month | 1.99 | 10,11 | 0.16 |
|  | Year | 0.41 | 10,11 | 0.52 |
|  | Year*Habitat breadth | 14.25 | 11,12 | 0.0002 |
|  | Month*Habitat breadth | 0.03 | 12,13 | 0.85 |
|  | Month*Year | 0.27 | 13,14 | 0.60 |
|  | Month*Year*Habitat breadth | 0.11 | 14,15 | 0.74 |
