# Supplementary material for: Long-term monitoring reveals an avian species credit in secondary forest patches of Costa Rica
Source: PeerJ. 2017 Jun 30;5:e3539. doi: 10.7717/peerj.3539 (PMC5494173; doi:10.7717/peerj.3539)
Supplement: Supplemental Information 5 [file peerj-05-3539-s005.docx]

Supporting Information, Appendix S5

Log-likelihoods and AIC values for multilevel Poisson-normal models with different traits as predictors of trends in net captures. There are two subsets of models: those that used data from both August and January, and one that only used data from January. Models that included both August and January data are of the form:

Null: N = **β_Int_.**

Year: N = **β_Int_.+ β_Year_***Year

Year+Trait: N = **β_Int._+ β_Trait_***Trait_SF_ **+ β_Jan_***Jan._Yes/No_ **+ β_Year_***Year

Year*Trait: N = **β_Int_.+ β_Trait_***Trait_SF_ **+ β_Jan_***Jan._Yes/No_ **+ β_Year_***Year **+ β_Year_*trait *Year***Trait_SF_

Where N is the number of birds captured in mist nets, Trait_SF_ is a 0/1 indicator for factor levels typical of primary (0) or secondary (1) forest (SF). Jan._Yes/No_ is an indicator variable for net captures in August (0) or January (1). Since latitudinal migrants occur only in January, these model lacked the **β_Jan_ *** Jan._Yes/No_ term. For both subsets of models with report AIC and log likelihoods for null models with no fixed effects and models with just year as a predictor to represent an overall trend for all species. For each trait we then report AIC and log likelihoods for the focal model with the Year*Trait interaction and a nested model that lacks this interaction (Year+Trait). We then report the AIC of a given model minus AIC of the appropriate null model (AIC-AIC_null_) to gauge whether inclusion of a trait in a model improves the model; negative values indicate improvements in the fit of the model relative to the null. P-values from Table 3 for the focal Year*trait interaction are also shown for reference.

| **Months** | **Trait** | **Model type** | **df** | **log Likelihood** | **AIC** | **AIC-AIC_null_** | **p-values** |
| --- | --- | --- | --- | --- | --- | --- | --- |
| **January** | Null model (no predictors) | null | 6 | -1031.4 | 2074.9 |  |  |
|  | Year model | year | 7 | -1031.4 | 2076.7 | -2.9 |  |
|  |  |  |  |  |  |  |  |
|  | Migration status | year+trait | 8 | -1031.3 | 2078.5 | -4.7 |  |
|  |  | year*trait | 9 | -1030.0 | 2078.0 | -4.1 | 0.11 |
|  |  |  |  |  |  |  |  |
| **August and January** | Null model (no predictors) | null | 8 | -2098.5 | 4213.0 |  |  |
|  | Year model | year | 9 | -2098.3 | 4214.6 | 1.6 |  |
|  |  |  |  |  |  |  |  |
|  | Habitat preference | year+trait | 11 | -2094.5 | 4211.0 | -2.1 |  |
|  |  | year*trait | 12 | -2088.8 | 4201.7 | -11.4 | 0.001 |
|  | Sensitivity to disturbance | year+trait | 11 | -2095.8 | 4213.6 | 0.5 |  |
|  |  | year*trait | 12 | -2085.7 | 4195.4 | -17.6 | <0.0001 |
|  | Conservation priority | year+trait | 11 | -2097.0 | 4216.1 | 3.0 |  |
|  |  | year*trait | 12 | -2096.1 | 4216.2 | 3.2 | 0.52 |
|  | Elevational migrant | year+trait | 11 | -2096.3 | 4214.6 | 1.6 |  |
|  |  | year*trait | 12 | -2096.1 | 4216.2 | 3.2 | 0.27 |
|  | Obligate Canopy Use | year+trait | 11 | -2097.3 | 4216.6 | 3.5 | 0.18 |
|  |  | year*trait | 12 | -2096.7 | 4217.3 | 4.3 |  |
|  | Canopy Use | year+trait | 11 | -2097.3 | 4216.6 | 3.5 |  |
|  |  | year*trait | 12 | -2097.1 | 4218.2 | 5.1 | 0.52 |
|  | Foraging guild-2 levels | year+trait | 11 | -2096.4 | 4214.8 | 1.8 |  |
|  |  | year*trait | 12 | -2094.3 | 4212.7 | -0.4 | 0.042 |
|  | Foraging guild-3 levels | year+trait | 12 | -2094.3 | 4212.6 | -0.4 |  |
|  |  | year*trait | 14 | -2091.8 | 4211.7 | -1.4 | 0.085 |
|  | Habitat breadth | year+trait | 11 | -2097.3 | 4216.6 | 3.5 |  |
|  |  | year*trait | 12 | -2090.2 | 4204.3 | -8.7 | 0.0002 |
