# Supplementary material for: Long-term monitoring reveals an avian species credit in secondary forest patches of Costa Rica
Source: PeerJ. 2017 Jun 30;5:e3539. doi: 10.7717/peerj.3539 (PMC5494173; doi:10.7717/peerj.3539)
Supplement: Supplemental Information 6 [file peerj-05-3539-s006.docx]

Supporting Information, Appendix S6

Slopes of trends for species with different traits calculated from Poisson-normal generalized linear mixed models. Slopes are on untransformed Poisson link scale and were calculated from marginal regression coefficients as necessary. Standard errors (SE) and p-values were calculated using the R package *multcomp*. SF = traits typical of species that primarily use secondary forest; MF = traits typical of species primarily using primary forest. (**) indicates p<0.05; (*) indicates 0.1> p>0.05.

| **Trait** | **Trait value** |  | **Slope** | **SE** | **p** |  |
| --- | --- | --- | --- | --- | --- | --- |
| **Migration  status** | Resident |  | 0.02 | 0.02 | 0.32 |  |
|  | Migrant | SF | -0.05 | 0.04 | 0.21 |  |
| **Habitat preference** | Primary forest | MF | 0.045 | 0.020 | 0.026 | ** |
|  | Second. forest | SF | -0.041 | 0.023 | 0.074 | ***** |
| **Disturbance sensitivity** | High or Medium | MF | 0.067 | 0.021 | 0.001 | ** |
|  | Low | SF | -0.043 | 0.021 | 0.038 | ** |
| **Conservation priority** | Medium | MF | 0.061 | 0.074 | 0.409 |  |
|  | Low | SF | 0.010 | 0.018 | 0.571 |  |
| **Elevational migrant** | Elev. mig | MF | 0.043 | 0.032 | 0.188 |  |
|  | Not elev. mig | SF | 0.005 | 0.019 | 0.786 |  |
| **Canopy use: obligate** | Obligate | MF | 0.068 | 0.045 | 0.127 |  |
|  | Fac. or Not used | SF | 0.007 | 0.018 | 0.720 |  |
| **Canopy use: any** | Oblig. or Fac. | MF | -0.001 | 0.026 | 0.975 |  |
|  | Not used | SF | 0.017 | 0.020 | 0.385 |  |
| **Foraging guild** | Specialist | MF | 0.027 | 0.019 | 0.170 |  |
|  | Omnivore | SF | -0.033 | 0.028 | 0.233 |  |
