# Supplementary material for: Long-term monitoring reveals an avian species credit in secondary forest patches of Costa Rica
Source: PeerJ. 2017 Jun 30;5:e3539. doi: 10.7717/peerj.3539 (PMC5494173; doi:10.7717/peerj.3539)
Supplement: Supplemental Information 7 [file peerj-05-3539-s007.docx]

Supporting Information, Appendix S7

Regression coefficients for models plotted in Figure 4. Models are from Poisson-normal generalized linear mixed models and are on the untransformed Poisson link scale. **β_Year_** and **β_Year*trait_** coefficients are combined to calculate realized trends as reported in Appendix S4 and are the basis for Figure 4. Models are of the form:

Net captures = **β_Int._+ β_Trait_** *Trait_SF_ **+ β_Jan_ ***Jan._Yes/no_ **+ β_Year_***Year **+ β_Year*trait_ *Year*** Trait_SF_

where Trait_SF_ is a 0/1 indicator for factor levels typical of primary (0) or secondary (1) forest (SF). Jan._Yes/no_ is an indicator variable for net captures in August (0) or January (1).

| **Model** | **β_Int._** | **SE** | **β_Trait_** | **SE** | **β_Jan._** | **SE** | **β_Year_** | **SE** | **β_Year*trait_** | **SE** |
| --- | --- | --- | --- | --- | --- | --- | --- | --- | --- | --- |
| Habitat preference | 0.25 | 0.15 | 0.50 | 0.23 | -0.18 | 0.13 | 0.04 | 0.02 | -0.09 | 0.02 |
| Sensitivity to dist. | 0.26 | 0.17 | 0.32 | 0.22 | -0.18 | 0.13 | 0.07 | 0.02 | -0.11 | 0.02 |
| Conservation priority | -0.28 | 0.52 | 0.73 | 0.53 | -0.19 | 0.13 | 0.06 | 0.07 | -0.05 | 0.07 |
| Elevational migrant | 0.38 | 0.29 | 0.04 | 0.31 | -0.20 | 0.13 | 0.04 | 0.03 | -0.04 | 0.03 |
| Canopy Use-Obligate? | 0.20 | 0.34 | 0.25 | 0.35 | -0.20 | 0.13 | 0.07 | 0.04 | -0.06 | 0.04 |
| Canopy Use-Any? | 0.45 | 0.22 | -0.05 | 0.25 | -0.19 | 0.13 | 0.00 | 0.03 | 0.02 | 0.03 |
| Foraging guild-2 | 0.67 | 0.25 | -0.33 | 0.27 | -0.19 | 0.13 | -0.03 | 0.03 | 0.06 | 0.03 |
| Habitat breadth | 0.43 | 0.30 | 0.00 | 0.08 | -0.19 | 0.13 | 0.12 | 0.03 | -0.04 | 0.01 |
