# Supplementary figures and images for: Long-term monitoring reveals an avian species credit in secondary forest patches of Costa Rica
Source: PeerJ. 2017 Jun 30;5:e3539. doi: 10.7717/peerj.3539 (PMC5494173; doi:10.7717/peerj.3539)

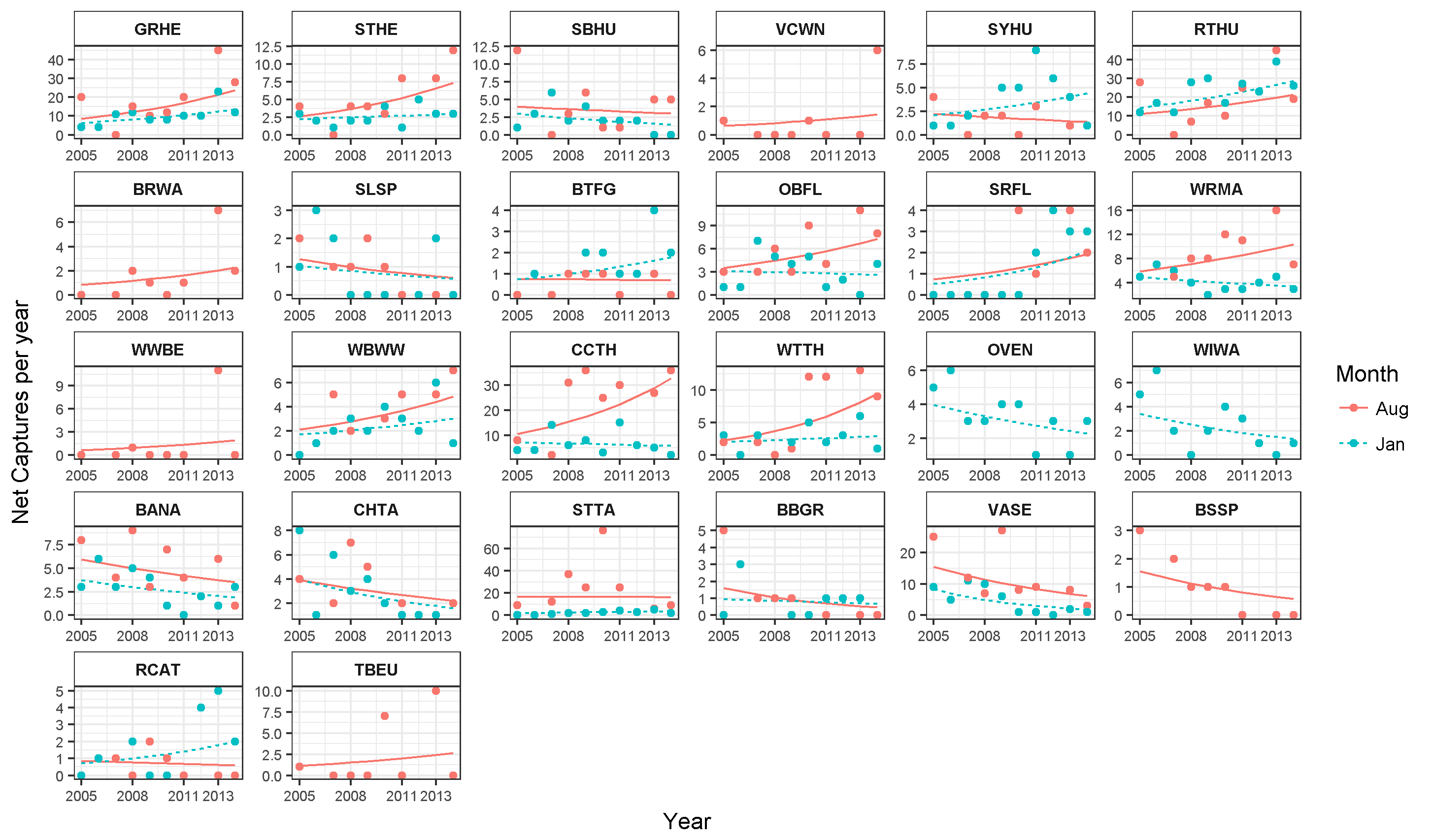

Supplement: Supplemental Information 8 [file peerj-05-3539-s008.png]
